# The Task Dependency of Spontaneous Rhythmic Performance in Movements Beyond Established Biomechanical Models: An Inertial Sensor-Based Study

**DOI:** 10.3390/s25216565

**Published:** 2025-10-24

**Authors:** Analina Emmanouil, Fani Paderi, Konstantinos Boudolos, Elissavet Rousanoglou

**Affiliations:** School of Physical Education and Sport Science, National and Kapodistrian University of Athens, 172 37 Daphne, Greece; fanipant@phed.uoa.gr (F.P.); cbountol@phed.uoa.gr (K.B.); erousan@phed.uoa.gr (E.R.)

**Keywords:** spontaneous motor tempo, physical fitness exercises, temporal structure, performance stability, variability

## Abstract

**Highlights:**

**What are the main findings?**

- Spontaneous motor tempo (SMT) is task-dependent: Complex, full-body exercises have slower and more variable tempos compared to simple, established movements like walking, hopping, and tapping.
- Consistent Relative Timing: Despite variations in overall tempo, the relative duration of a movement cycle’s phases remains stable, typically at a 1:1 ratio. This points to a shared, underlying neural control mechanism.
- High Performance Stability: Individuals maintain a high degree of timing precision with low individual variability, even during complex, full-body exercises.

**What are the implications of the main findings?**

- Internal timing mechanisms are adaptable and adjust to task-specific biomechanical demands, providing insights into motor control beyond simple rhythmic movements.
- The findings provide a basis for designing personalized exercise and rehabilitation programs that account for an individual’s natural, task-specific rhythmic variability.

**Abstract:**

Spontaneous rhythmic performance is a fundamental feature of human movement, well established in biomechanical models (EBMs) but less understood in complex physical fitness exercises (PFEs). This study examined the task dependency of spontaneous rhythmic performance across three EBMs (walking, hopping, finger tapping) and seven PFEs (hip abduction, back extension, sit-up, push-up, shoulder abduction, squat, lunge). A total of 15 men and 15 women performed each task at a self-selected pace while wearing inertial sensors. Measures included spontaneous motor tempo (SMT), temporal structure metrics, and their within- and between-trial individual variability (%CV) (ANOVA, SPSS 28.0, *p* ≤ 0.05). SMT was task-dependent, with EMB tasks being near ~2 Hz (walking: 1.82 ± 0.10 Hz; hopping: 2.08 ± 0.22 Hz; finger tapping: 1.89 ± 0.43 Hz) and PFEs being slower (0.36–0.68 Hz). Temporal structure mirrored these differences with shorter cycle and phase durations in EBM than PFE tasks, with relative phase durations consistently at about a 1:1 ratio. Τhe overall low %CV indicated stable performance (within-trial: 1.4–7.5%; between-trial: 0.5–7.8%). The results highlight the task dependency of SMT and temporal structure, as well as the robustness of an overarching internal timing framework supporting rhythmic motor control across diverse movement contexts.

## 1. Introduction

Spontaneous rhythmic performance is a fundamental characteristic of many human behaviors. From walking and clapping to dancing or fitness exercise, rhythmic movement structures emerge—sometimes inherently, other times through practice and repetition. In the absence of external pacing cues, these rhythmic performances often arise spontaneously, shaped by the interplay of internal neural timing systems and biomechanical constraints. At the heart of this phenomenon lies the concept of spontaneous motor tempo (SMT), referring to an individual’s naturally preferred frequency for performing repetitive movements without external pacing stimuli [1,2,3,4,5,6,7,8,9].

Traditionally, SMT has been examined in simple tasks such as finger tapping, foot tapping, spot stepping, hopping, and walking—motor tasks characterized by rhythmic clarity and mechanical simplicity, ideal for isolating endogenous timing mechanisms. Walking is a whole-body, cyclic behavior partly governed by spinal cord pattern generators and neural oscillators [10,11,12], while tapping relies more on voluntary discrete motor control and cognitive timing mechanisms [12,13,14], though repeated tapping can also recruit lower-level neural circuitry [15]. Despite these differences in underlying mechanisms, SMT across such tasks tends to converge around a frequency near 2 Hz [2,3,4,8,11,13,14,15,16,17,18,19,20,21], suggesting the existence of a default rhythmic tendency—as proposed in human resonance theory [18].

Spontaneous motor tempo (SMT) exhibits high temporal stability at the intra-individual level, as evidenced by low variability across different time points [1,4,5,6,7,8,19,22]. At the same time, considerable inter-individual differences have been reported, as reflected in the broad variation in SMT values across individuals [1,4,7,20,22]. For instance, the finger tapping SMT has been observed within the broad range of 0.3–5 Hz [1,20,22], challenging the idea of a single universal tempo. Instead, SMT may be better conceptualized as a probabilistic tendency shaped by factors such as age, arousal, fatigue, or musical training [1]. The intra-individual stability of SMT, in turn, reflects a flexible but robust internal timing architecture that enables rhythmic movement even under varying task demands [1,2,7,9,16,23].

Biomechanical constraints—such as limb mass, joint mobility, and mechanical load—further shape SMT by influencing the energetically optimal tempo for different tasks [1,7,20]. Movements involving heavier or multi-joint segments tend to adopt slower tempos due to increased mechanical demands [24,25,26]. These constraints do not eliminate variability; instead, they structure it into functional flexibility, allowing the timing system to adapt while remaining bounded by biomechanical limits [9]. Consequently, SMT at the group level may vary significantly across movements depending on biomechanical complexity, familiarity, or automatization.

Despite growing interest in SMT, research has primarily focused on inherently rhythmic or well-practiced tasks, such as walking, hopping, and tapping. Little is known about the spontaneous timing of more complex, voluntary movements—such as physical fitness exercises (PFEs)—which are not inherently rhythmic but can acquire a cyclic temporal structure through repetition. These tasks involve increased biomechanical and muscular complexity, which may affect both SMT and temporal performance stability. Although current theoretical and empirical frameworks imply that SMT is task-dependent [1,7,20,24,27], to the best of our knowledge, no studies have examined how SMT manifests in voluntary movements, particularly those with increased biomechanical and muscular demands, that fall outside established biomechanical rhythmic models. This critical gap in our understanding raises key questions:

- Do individuals maintain an SMT during voluntary physical fitness exercise tasks performed without external pacing?
- How does SMT in such exercise tasks compare to that observed in established biomechanical model (EBM) tasks regarding temporal structure metrics and stability?

Answering these questions is not only of theoretical importance but also important for informing strategies to optimize exercise performance, mitigate injury risks, and enhance overall exercise experiences. Recognizing differences in preferred temporal patterns and their stability is crucial in exercise programs and sports training, where rhythmic temporal cues are often used to guide timing, enhance performance, and improve movement accuracy.

One key limitation in past SMT studies has been methodological: traditional motion capture systems are often unsuitable for naturalistic, whole-body movements. In contrast, wearable inertial measurement units (IMUs) provide a lightweight, cost-effective, and scalable alternative. These sensors can accurately capture kinematic and temporal data during dynamic, real-world movement [28,29]. Even a single IMU, when placed on a representative body segment, can reliably detect angular velocity patterns and extract key rhythmic features—such as cycle duration, absolute and relative phase durations, and overall tempo—even with just a few movement repetitions [28,29]. Their reliability makes them well-suited for capturing spontaneous motor timing during both simple and complex motor tasks [29].

This study aimed to investigate the task dependency of spontaneous motor performance across a range of movement tasks—from established biomechanical models to voluntary, physical fitness-based exercise tasks characterized by increased biomechanical, muscular, and postural demands.

## 2. Materials and Methods

### 2.1. Participants

Thirty active men (*n* = 15) and women (*n* = 15) participated in this study. The male participants had an average age of 24.8 ± 3.3 years, body height of 1.8 ± 0.1 m, body mass of 80.2 ± 8.5 kg, and BMI of 24.3 ± 2.1 kg/m^2^. The female participants had an average age of 28.9 ± 6.6 years, body height of 1.7 ± 0.04 m, body mass of 59.3 ± 6.3 kg, and BMI of 21.8 ± 2.0 kg/m^2^. All participants had various sports backgrounds, including basketball, football, gymnastics, and weightlifting, with at least two years of strength training experience. All were right-handed and right-footed, physically active (exercising at least three times per week for the past six months with sessions lasting at least 60 min), and familiar with the movement patterns assessed in this study. Inclusion criteria required no history of vestibular, orthopedic, or neurological disorders in the past 12 months, as these could affect their movement patterns. A priori power analysis (G*Power 3.1) for a repeated-measures ANOVA (within–between interaction: 10 movement tasks × 2 groups) indicated that a minimum total sample of N = 20 (10 males and 10 females) would be required (η^2^ = 0.16; α = 0.05; power = 0.95; ε = 1; actual power = 96%). Therefore, our final sample of N = 30 exceeds this requirement and provides adequate statistical power for the planned analyses. This study was approved by the Bioethics Committee of the School of Physical Education and Sports Science, National and Kapodistrian University of Athens, Greece (approval ref. No. 1354/03/03/2022). Written informed consent was obtained from all participants in accordance with the principles of the Helsinki Declaration.

### 2.2. Experimental Procedure

Participants performed ten different rhythmic movement tasks at their preferred, most comfortable tempo. These included 3 EBMs (hopping, finger tapping, and walking), as well as 7 PFEs (hip abduction, back extension, sit-up, push-up, shoulder abduction, squat, and lunge). For each task, participants completed three trials of 20 consecutive repetitions each. Two minutes of rest was provided between-trials of the same task to prevent fatigue.

The order of tasks was randomized using a rotation sequence: the sequence order was fixed, but each participant started with the movement following the initial task performed by the previous participant. Instructions were given both verbally and visually before data collection to ensure proper technique [29]. Throughout each trial, participants were visually monitored by the same examiner who had previously evaluated their body form during the familiarization session. All measurements took place in the morning between 9:00 and 12:00 to control circadian influences [2,22].

#### 2.2.1. Data Collection and Analysis

**Figure 1:**
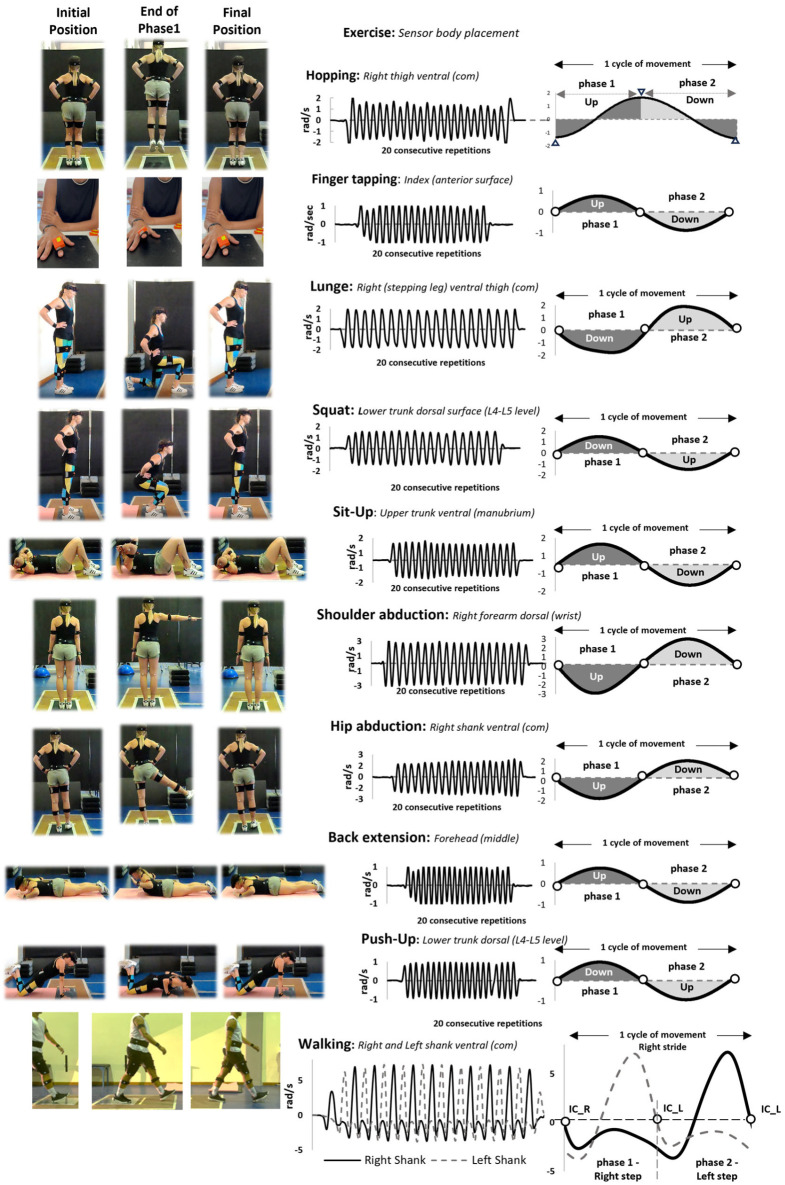
Exemplary angular velocity trajectories (**middle**) in each movement pattern (**left**, the sensor position on the body is noted). The cycle duration (**right**) is schematically defined as the time taken between 2 consecutive zero-crossing points (circle markers), indicating the same directional change. For the two-leg hop only, the cycle duration is defined between two consecutive dips (triangle markers). In walking, this cycle duration corresponds to the stride duration of a given leg, while the step duration (phases) is defined as the time between the zero-crossing of one shank and the subsequent zero-crossing of the contralateral shank.

Inertial sensors (Xsens MTw Awinda; Movella, Henderson, NV, USA; sampling at 100 Hz, including a triaxial accelerometer, gyroscope, and magnetometer) were placed on participants’ body segments to record angular velocity trajectories. The sensors were secured with elastic straps to minimize movement artifacts. A total of six sensors were placed on task-relevant body segments. For analysis, one sensor was selected for each exercise, corresponding to the segment that exhibited the greatest range of motion in the primary movement plane. This approach ensured the extraction of the most representative angular velocity trajectory for each task (Figure 1) [29].

The angular velocity trajectories displayed clear periodic waveforms with positive and negative regions corresponding to the two movement phases. Key events such as initiation, termination, and change in direction were identified using zero-crossing points, peaks, and dips. Before the extraction of temporal metrics, a 4th-order Butterworth low-pass filter with a cut-off frequency of 2 Hz was applied to all angular velocity signals to remove high-frequency noise while preserving the primary movement signal (MATLAB R2022b, MathWorks, Inc., Natick, MA, USA). The cut-off frequency was determined by examining the frequency power spectrum of all raw signals across tasks and participants, which consistently showed the highest signal intensity below 2 Hz across all tasks (even for those with an inherent movement cycle frequency below 1 Hz) and across all participants (Appendix E—Figure A2). The frequency spectrum was computed using FFT analysis on the raw angular velocity signals, and the filter was applied in MATLAB R2022b using zero-phase filtering to avoid phase distortion [29].

#### 2.2.2. Extraction of Spontaneous Motor Performance Variables

##### Temporal Structure Metrics

The movement cycle and its two phases (phase1 and phase2) were defined by the zero-crossing points of angular velocity trajectories. Each cycle consisted of three zero-crossing points: start (1st), midpoint (2nd), and end (3rd). Depending on sensor orientation, cycles were defined from positive to negative zero-crossings (walking, finger tapping, hip abduction, shoulder abduction, lunge) or from negative to positive (back extension, sit-up, push-up, squat). Hopping cycles were defined as intervals between two consecutive dips [29].

Cycle duration was calculated as the time interval between two consecutive zero-crossing points in the same direction. Phase1 was the time from the 1st to the 2nd zero-crossing point, while phase2 was the time from the 2nd to the 3rd zero-crossing point. These time intervals were used to calculate absolute movement durations (tcycle, tphase1, tphase2, in s) and relative movement durations (%tphase1, %tphase2, as percentages of tcycle). Zero-crossing points were detected using MATLAB’s zero-crossing detection technique, and the peak detection technique was used to identify peaks and dips in the two-leg hop series (MATLAB R2022b, MathWorks, Inc., Natick, MA, USA).

For each participant, the mean of 15 selected repetitions was calculated per trial, and the average of three trials was computed to obtain the final temporal structure metrics. These values were used in the subsequent statistical analyses. Group-level variation was assessed to examine homogeneity within the group. The coefficient of variation expressed as a percentage (CV%) was used to quantify the group-level variation. Group-level CV% was defined as the ratio of the group standard deviation to the group mean (SD/Mean) multiplied by 100.

The SMT was calculated per participant using the formula f = 1/T (Hz), where T represents the mean cycle duration (in s) averaged over the selected repetitions and trials. Thus, SMT (in Hz) reflects the participant’s naturally preferred movement frequency based on the mean cycle duration extracted from their performance [1,2,4,5,6,7,8,9].

##### Temporal Structure Stability

Temporal stability was evaluated along two dimensions of individual variability for all temporal structure metrics: within-trial and between-trial.

Within-trial individual variability was calculated as the coefficient of variation (CV) across repetitions within a single trial:

(1) $${CV}_{15reps}=\frac{SD_{15reps}}{Mean_{15reps}}\times 100$$

where SD15reps and Mean15reps specifically represent the standard deviation and mean of the 15 consecutive repetitions within a trial. The average of the three trials’ CV_15reps_ was used to represent each participant’s within-trial variability.

Between-trial individual variability was calculated as the coefficient of variation across repeated trials of the same task:

(2) $${CV}_{3trials}=\frac{SD_{3trials}}{Mean_{3trials}}\times 100$$

where SD3trials and Mean3trials represent the standard deviation and mean across the three trial-level averages. This single CV3trials represented each participant’s between-trial variability.

In both cases, individual variability was expressed as a percentage (CV%), defined as CV × 100.

##### Selected Cycles and Reliability Assessment

Prior work indicates that reliable temporal estimates can be obtained with relatively few repetitions for the selected tasks [29]. Other studies have reported using 6–40 repetitions or trial durations between 12 and 120 s for EBM tasks, but these protocols are not directly applicable to the PFEs used here [1,3,4,17,25]. To ensure both reliability and comparability with these biomechanical models, 15 repetitions were analyzed per trial, after excluding the first and last repetitions to minimize initiation and termination effects. For push-ups, only 10 central repetitions were included due to participants’ maximum successful repetitions being reached. The selection of 15 repetitions is supported by prior work demonstrating reliable temporal estimates with fewer repetitions [29]. All selected repetitions were visually and statistically screened for outliers in the cycle duration variable (tcycle), as this was the primary variable of interest for calculating temporal structure metrics and spontaneous motor tempo. Outliers in tcycle were identified per participant and task using the 1.5 × IQR boxplot criterion using the Explore procedure in SPSS and replaced using mean imputation based on the remaining repetitions within the same participant and task. This approach preserves participant-specific temporal characteristics while maintaining dataset integrity. Only 20 cycles (0.15% of the dataset) required imputation, ensuring minimal influence on the results.

The choice of 15 repetitions was supported by prior research demonstrating reliable estimates even with fewer repetitions [29]. However, a larger number of repetitions was selected here to better capture temporal stability across cycles. To confirm the reliability of these 15 repetitions, test–retest reliability indices were computed:

(a) The Intraclass Correlation Coefficient (ICC) using a two-way random-effects model for absolute agreement and average measures.
(b) The Standard Error of Measurement (SEM) derived from the square root of the ANOVA residual mean square error (SEM = √S^2^error) and its relative form expressed as a percentage of the mean (SEM% = (SEM/$\bar{x}$) × 100).
(c) The Minimal Detectable Change at 95% confidence (MDC95), calculated from the SEM as MDC95 = SEM × 1.96 × √2, and its relative form, expressed as a percentage of the mean (MDC95% = (MDC95/$\bar{x}$) × 100).

These reliability indices are presented in Appendix D: Table A7 (ICC), Table A8 (SEM and SEM%), and Table A9 (MDC95 and MDC95%). Collectively, reliability analysis confirmed an ICC ≥ 0.90 and SEM ≤ 5%, demonstrating the consistency and robustness of the temporal structure metrics across the 15 retained repetitions. The observed high ICC values (≥0.90) and low SEM% values (≤5%), along with MDC95% values that consistently exceed the upper boundary of the SEM, indicate strong agreement across repetitions. This confirms that the selected repetitions provide reliable and reproducible measurements for the assessed movement tasks.

#### 2.2.3. Statistical Analysis

Descriptive statistics (mean, SD) were calculated for all temporal structure metrics (tcycle, tphase1, tphase2, %tphase1, %tphase2) and their stability (within-trial and between-trial individual %CVs). Normality assumptions were checked via the Shapiro–Wilk test, skewness, kurtosis, and standardized z-scores, confirming parametric testing’s suitability [30]. A two-way mixed-design ANOVA was performed to examine the effects of movement task (within-subjects factor) and gender (between-subjects factor: male vs. female) on the temporal metrics. No significant interaction was observed between the rhythmic movement task and gender for any of the temporal metrics (*p* > 0.05); therefore, the results are presented and interpreted for the combined sample regardless of gender. Post hoc pairwise comparisons with Bonferroni correction were conducted following significant main effects. The sphericity assumption was assessed using Mauchly’s test, with the Greenhouse–Geisser correction applied when sphericity was violated. Effect sizes were reported as partial eta squared (η^2^), interpreted as 0.01 = small, 0.06 = medium, and ≥0.14 = large [31]. Statistical significance was set at *p* < 0.05. All analyses were conducted using SPSS Statistics (Version 29.0; IBM Corp., Armonk, NY, USA).

## 3. Results

### 3.1. Task Dependency of Spontaneous Motor Tempo

**Figure 2:**
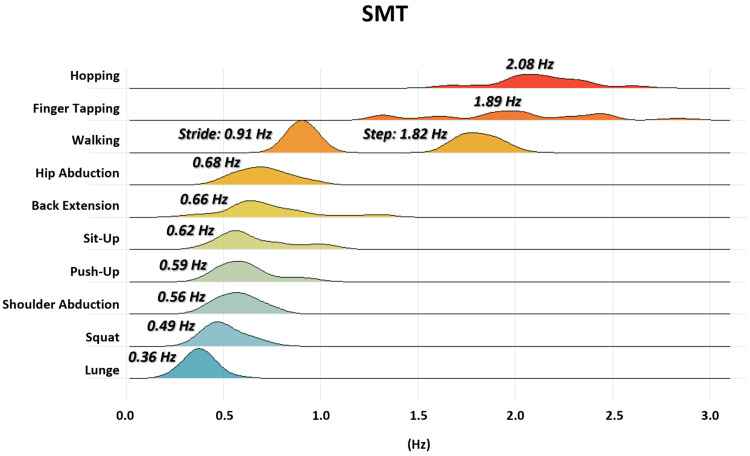
A density ridge plot displaying the range of spontaneous motor tempo (Hz) in the three EBM and seven PFE rhythmic movement tasks, across the 30 participants, calculated from the average of the final 15 repetitions (mean of 3 trials per subject). Above each density curve, the respective mean motor tempo is indicated.

Spontaneous motor tempο (SMT) significantly differed between movement tasks (*p* ≤ 0.001), as reflected by movement frequency (Hz) (Figure 2). The EBM tasks clustered near ~2 Hz, consistent with previous reports, with hopping (2.08 ± 0.22 Hz), finger tapping (1.89 ± 0.43 Hz), and walking (1.82 ± 0.10 Hz when calculated by step frequency, 0.91 ± 0.05 Hz when calculated by stride frequency) ranking among the fastest.

In contrast, all PFE tasks presented significantly lower tempos, ordered from faster to slower (Figure 2) as follows: hip abduction (0.68 ± 0.13 Hz), back extension (0.66 ± 0.22 Hz), sit-up (0.62 ± 0.16 Hz), push-up (0.59 ± 0.12 Hz), shoulder abduction (0.56 ± 0.10 Hz), squat (0.49 ± 0.09 Hz), and lunge (0.36 ± 0.07 Hz).

Pairwise comparisons further revealed significant differences among PFE tasks, particularly between faster (hip abduction, back extension, sit-up, push-up) and slower tasks (squat, lunge) (Figure 2).

### 3.2. Task Dependency of Temporal Structure

**Figure 3:**
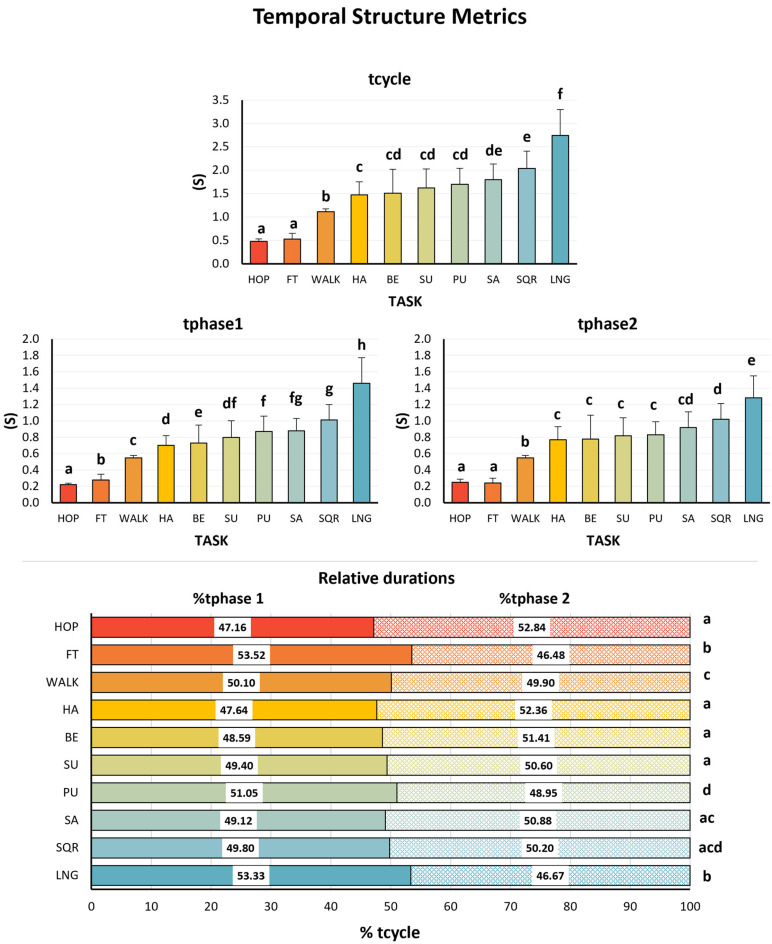
Τemporal structure metrics (mean ± SD) for each of the three EBM and seven PFE rhythmic movement tasks. Absolute durations—tcycle (**top** panel), tphase1, and tphase2 (**middle** panels)—and relative durations—%*tphase1 and %tphase 2* (**bottom** panel). Each bar represents the mean value for a given task, with error bars indicating the standard deviations. Different letters above the bars indicate statistically significant differences between tasks based on post hoc comparisons (Compact Letter Display; *p* < 0.05). Tasks sharing the same letter do not differ significantly. HOP: hopping; FT: finger tapping; WALK: walking; HA: hip abduction; BE: back extension; SU: sit-up; PU: push-up; SA: shoulder abduction; SQR: squat; LGN: lunge.

The metrics of temporal structure differed significantly across movement tasks (*p* < 0.001), with large effect sizes (partial η^2^ = 0.50–0.93). These values indicate that 50% to 93% of the variance in temporal metrics was explained by task type, far exceeding the conventional threshold for a large effect (≥0.14). Cycle duration (tcycle) was systematically shorter in EBM tasks, with hopping (0.48 ± 0.05 s), finger tapping (0.53 ± 0.12 s), and walking (0.55 ± 0.03 s per step cycle, 1.11 ± 0.06 s per stride cycle) showing the fastest tempos. In contrast, PFE tasks exhibited progressively longer cycle durations, from hip abduction (1.47 ± 0.28 s) to lunge (2.74 ± 0.56 s) (Figure 3, top panel). Absolute phase durations (tphase1, tphase2) followed the same pattern, being shorter in EBM tasks and longer in PFE tasks (Figure 3, middle panel).

Relative phase durations (%tphase1 and %tphase2) also differed significantly between tasks (*p* < 0.001), but differences were not systematically associated with task type (EBM vs. PFE). Walking and squat exhibited approximately equal %tphase (50–50%), while hopping, hip abduction, back extension, sit-up, and shoulder abduction showed slightly shorter %tphase1 (≈48%) and correspondingly longer %tphase2 (≈52%). In contrast, finger tapping, push-up, and lunge displayed longer %tphase1 (≈53%) and correspondingly shorter %tphase2 (≈47%) (Figure 3, bottom panel). Detailed numerical results and pairwise comparisons for all metrics are presented in Appendix A: Table A1 and Appendix B: Table A4.

**Figure A1:**
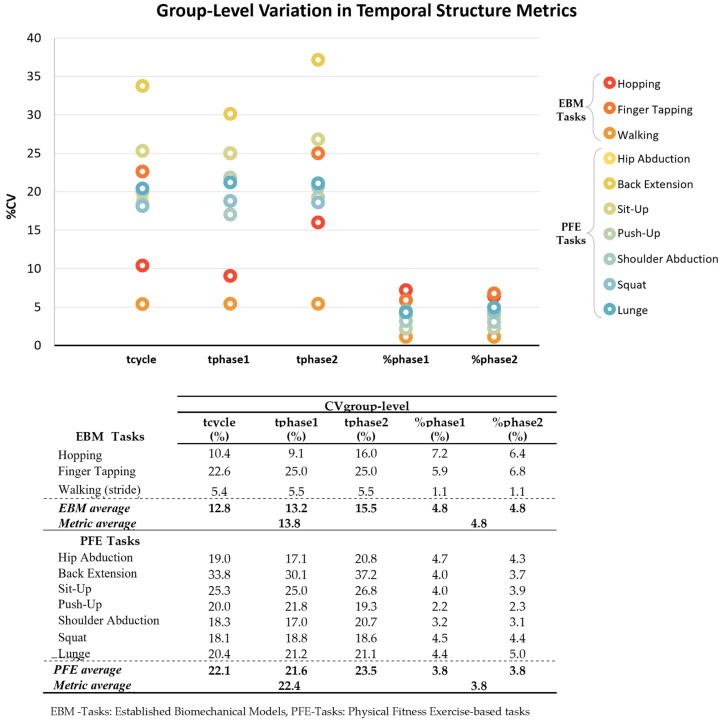
Group-level variation (CVgroup-level) in temporal structure metrics for the three EBM and seven PFE rhythmic movement tasks. Values are expressed as the percentage coefficient of variation (%CV), representing between-individual variability, where a higher %CV indicates lower group homogeneity. The graph (**top**) shows CVgroup values across tasks, while the table (**bottom**) provides detailed CV values for cycle duration (tcycle), absolute phase durations (tphase1, tphase2), and relative phase durations (%tphase1, %tphase2). EBM Average and PFE Average represent the mean %CV across all tasks within each task group, and Metric Average represents the mean CV across absolute durations (tcycle, tphase1, tphase2) and relative durations (%tphase1, %tphase2) within each task group.

**Figure A2:**
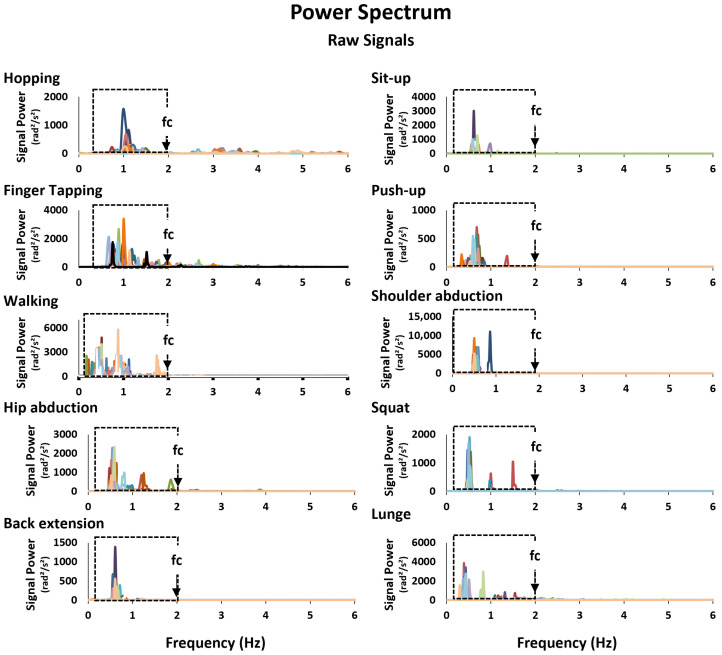
The power spectrum of angular velocity for 30 subjects in one trial across three EBM and seven PFE rhythmic movement tasks. The coloured lines represent the participants of the study. Signal power (proportional to squared amplitude) was computed using a discrete Fourier transform. The graphs illustrate the power spectrum (distribution of signal energy across different frequencies) before the application of a noise reduction filter (4th-order Butterworth low-pass filter; fc: cut-off frequency 2 Hz), corresponding to one representative trial; the same pattern was observed consistently across the other two trials. In all movement tasks, the raw signals prominently highlight the desired frequency range below 2 Hz. A 4th-order Butterworth low-pass filter (cut-off frequency: 2 Hz) was applied to reduce high-frequency noise while preserving this relevant range, thereby enhancing signal clarity for subsequent zero-crossing and peak-finding analyses. Power spectrum estimation followed the Basic Spectral Analysis in Matlab 2023b (https://www.mathworks.com/help/matlab/math/basic-spectral-analysis.html accessed on 1 October 2024).

A descriptive analysis of group-level variation (CVgroup) showed consistently greater between-participant variability across metrics in PFE tasks (avg ≈ 15%) compared to the two EBM tasks (walking and hopping avg ≈ 6.8%), with finger tapping showing comparably elevated variability (17.1%), aligning with the PFE average. CVgroup was the highest for cycle and absolute phase durations (avg 22.4% for PFE, 13.8% for EBM) and substantially lower for relative phase durations (avg 3.8% for PFE, 4.8% for EBM). Detailed CVgroup values are provided in Appendix C: Figure A1.

### 3.3. Task Dependency of Temporal Structure Stability

#### 3.3.1. Overall Variability Tendency Across Temporal Structure Metrics

**Figure 4:**
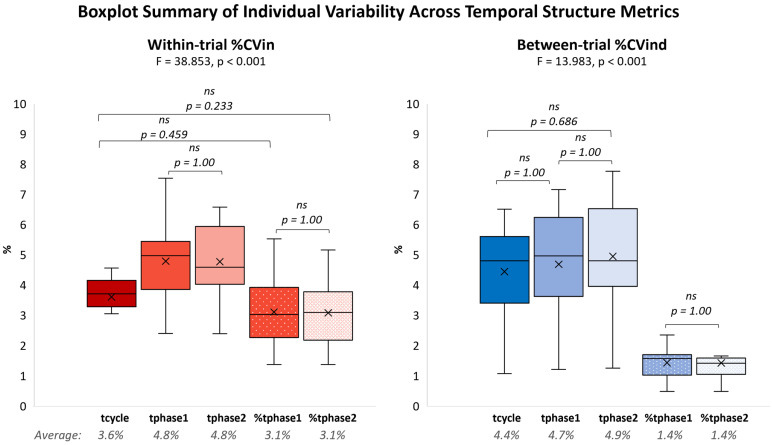
A summary of individual variability (%CV) across the three EBM and seven PFE rhythmic movement tasks for each temporal metric. **Left** panel: Within-trial (intra-trial) variability. **Right** panel: Between-trial (inter-trial) variability. Each boxplot summarizes the distribution of variability across all ten tasks for a given metric, illustrating the central tendency and spread of variability to highlight overall tendencies across metrics. Non-significant (ns) pairwise comparisons with corresponding *p*-values are noted above the bars.

*Across* all movement tasks, variability—both within-trial (repetition to repetition within the same single trial) and between-trial (trial to trial across repeated executions)—was consistently lower for relative phase durations (%tphase1, %tphase2) compared to absolute durations (tcycle, tphase1, tphase2) (Figure 4). Specifically, relative durations showed an average within-trial variability of ≈3.1% and an even smaller between-trial variability of ≈1.4%. In contrast, absolute durations exhibited higher variability, with within-trial values of ≈3.6% (tcycle), ≈4.8% (tphase1), and ≈4.8% (tphase2) and between-trial values of ≈4.4% (tcycle), ≈4.7% (tphase1), and ≈4.9% (tphase2).

**Figure 5:**
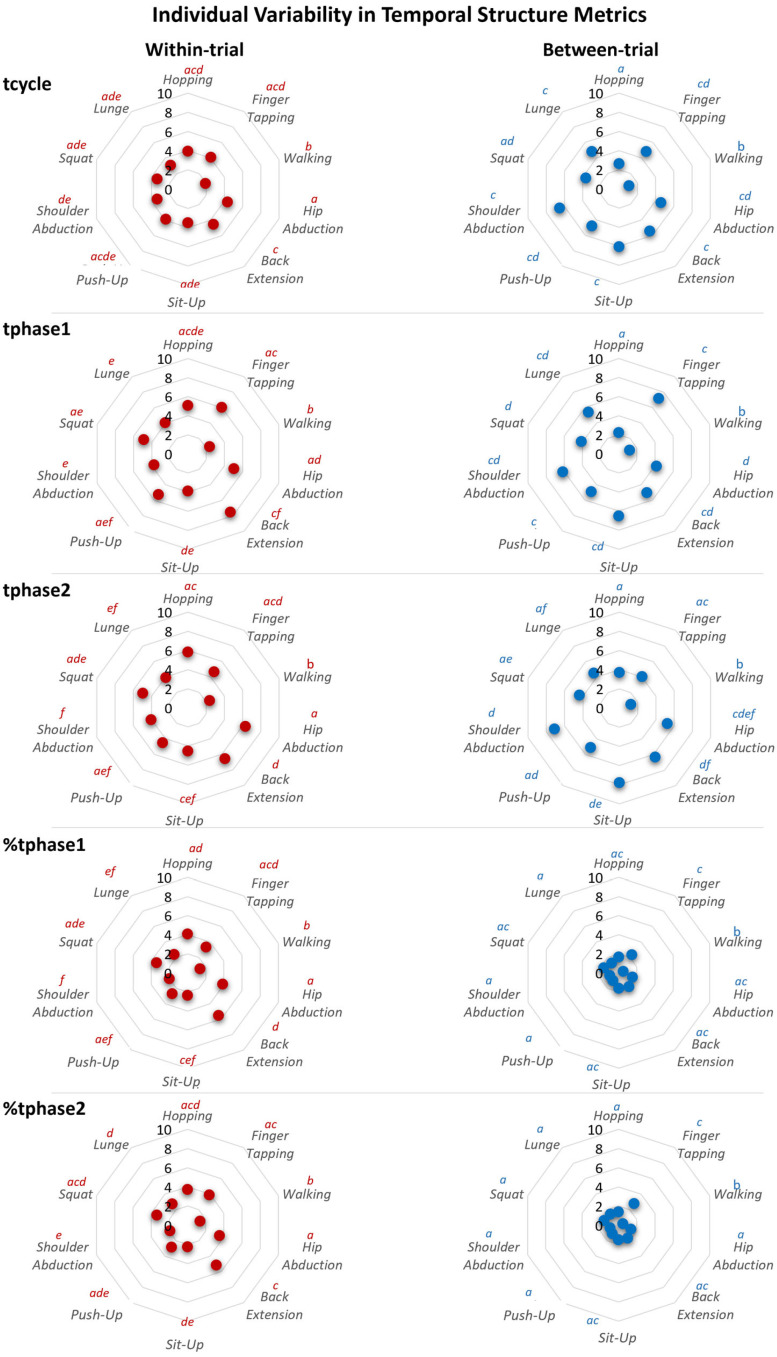
The mean individual variability (%CV) in rhythmic temporal structure metrics across the three EBM and seven PFE rhythmic movement tasks. Panels are organized by variability type (columns) and metric (rows): **Left** column—within-trial variability. **Right** column—between-trial variability. Rows represent the five temporal metrics: tcycle, tphase1, tphase2, %tphase1, and %tphase2. Different letters above the movement task labels indicate statistically significant differences between tasks based on post hoc comparisons (Compact Letter Display; *p* < 0.05). Tasks sharing the same letter do not differ significantly.

As analytically shown in Figure 5, temporal structure stability was statistically task-dependent, as both within-trial and between-trial individual variability differed significantly between tasks.

#### 3.3.2. Within-Trial Individual Variability

Within-trial variability differed significantly between tasks for all temporal structure metrics (*p* < 0.001), with large effect sizes (partial η^2^ = 0.34–0.38) (Figure 5, left panels). This indicates that approximately 34–38% of the variance in within-trial variability was attributable to task type, reflecting a strong influence of task characteristics on temporal stability at the cycle level.

Walking consistently exhibited the lowest within-trial variability across all metrics (tcycle ≈ 1.9%; tphase1 and tphase2 ≈ 2.4%; %tphase1 and %tphase2 ≈ 1.4%), compared to both PFE tasks and to the other two EBM tasks (hopping and finger tapping), indicating a highly stable temporal structure within-trial.

Among the PFE tasks, back extension showed the highest within-trial variability (ranging from 4.6% to 7.5% across metrics), differing significantly from most other tasks.

Detailed numerical results and pairwise comparisons for all metrics are presented in Appendix A: Table A2 and Appendix B: Table A5.

#### 3.3.3. Between-Trial Individual Variability

Between-trial variability differed significantly between tasks for all temporal structure metrics (*p* < 0.001), with large effect sizes (partial η^2^ = 0.34–0.38) (Figure 5, right panels). Thus, 34–38% of the variance in between-trial variability was explained by task type, confirming that movement characteristics strongly influenced stability across repeated executions.

Consistent with the within-trial results, walking exhibited the lowest between-trial variability across all metrics (tcycle ≈ 1.1%; tphase1 ≈ 1.2%; tphase2 ≈ 1.3%; %tphase1 and %tphase2 ≈ 0.5%), compared to both PFE tasks and to the other two EBM tasks (hopping and finger tapping), indicating a highly stable temporal structure between-trial.

Second to walking, the hopping EBM task also showed significantly lower between-trial variability compared with all other tasks (except walking) for absolute durations (tcycle ≈ 2.6%; tphase1 ≈ 2.2%; tphase2 ≈ 1.9%) but not for relative durations.

In contrast, the finger tapping EBM task displayed higher between-trial variability (≈2.4–7.2%) compared to walking, hopping EBM tasks, and several PFE tasks.

Detailed numerical results and pairwise comparisons for all metrics are presented in Appendix A: Table A3 and Appendix B: Table A6.

## 4. Discussion

This study aimed to investigate the task dependency of spontaneous motor performance across a range of movement tasks—ranging from EBM to voluntary PFE tasks characterized by increased biomechanical, muscular, and postural demands. Specifically, besides the SMT, it also examined rhythmic temporal structure metrics (cycle duration, absolute and relative phase durations), as well as their stability (assessed through within- and between-trial individual variability). This design enabled an evaluation of how SMT, temporal structure, and stability differ across tasks and how internal timing processes remain robust under varying demands.

The results revealed a clear task dependency of spontaneous motor performance. EBM tasks (walking, hopping, finger tapping) produced faster tempos clustered around ~2 Hz with correspondingly shorter cycle and absolute phase durations. In contrast, PFE tasks exhibited significantly slower tempos (0.36–0.68 Hz) and longer cycles and phase durations. The group-level variability in these temporal structure metrics (cycle and absolute phase durations) was lower in EBM tasks (avg CVgroup ≈ 13.8%) than in PFE tasks (avg CVgroup ≈ 22.4%), indicating greater participant consistency in the former.

Notably, despite these tempo and duration differences, the relative timing of movement phases (%tphase1 and %tphase2) remained close to a 1:1 ratio, with low group-level variability (CVgroup ≈ 4.8% and ≈ 3.8% for EBM and PFE, respectively), suggesting that relative phase durations can be considered functionally not task-dependent.

Temporal structure stability was task-dependent, with walking and hopping showing the lowest individual variability across metrics (within-trial avg CVind ≈ 3.2%; between-trial avg CVind ≈ 1.6%), indicating the greatest stability. Nevertheless, all tasks exhibited generally low variability: within-trial (avg CVind ≈ 3.9%) and between-trial (mean CVind ≈ 3.4%) remained consistently low, with no metric exceeding 7.8%. These results suggest that, although stability was the highest in the two EBM tasks, rhythmic temporal structure was robust across the full range of movement tasks.

*Task Dependency of Spontaneous Motor Tempo.* Participants exhibited distinct SMT values across the ten rhythmic movement tasks. The EBM tasks—two-leg hopping, finger tapping, and walking—yielded spontaneous tempos close to 2 Hz, which aligns with prior research on natural movement resonance [2,3,4,8,11,13,14,15,16,17,18,19,20,21]. For walking, however, this frequency corresponds to the step cycle (single footfall) rather than the full gait cycle (stride frequency, including both left and right steps), which occurs at approximately 1 Hz. This distinction reflects a fundamental measurement convention: while all other tasks in this study were evaluated over their full movement cycle, walking’s inherent forward-motion rhythm requires specifying whether the tempo refers to steps or strides [3,18].

In contrast, PFE tasks demonstrated slower SMT (0.36–0.68 Hz), most likely reflecting greater segmental inertia, muscular force requirements, and coordination demands [20,24,27]. The fastest PFE tasks (hip abduction and back extension) were nearly twice as fast as the slowest ones (squat and lunges), yet all remained well below the ~2 Hz typical of EBM tasks. This tempo spread highlights how movement task type and biomechanical constraints strongly shape SMT [1,7,20,24,27].

Although minimizing the energy cost is often considered a primary reason for spontaneous tempos, evidence from gait and cycling studies shows that individuals sometimes prefer tempos that feel comfortable or efficient, even if they are more energetically demanding [10]. This aligns with our finding that SMT is highly task-dependent, suggesting that spontaneous tempo may arise from intrinsic neural mechanisms—such as central pattern generators—that produce stable and individualized rhythmic outputs [10]. For our diverse PFE tasks, the notably lower SMTs compared to the 2 Hz of EBM tasks [2,3,4,8,11,13,14,15,16,17,18,19,20,21] likely reflect a combination of this internal timing system and the increased biomechanical and postural demands of PFEs, favoring slower, more controlled movement rhythms over tempos dictated solely by metabolic efficiency.

Direct comparison with prior work is challenging, as also highlighted in the systematic review by Desbernats and colleagues [1], due to heterogeneity in participant characteristics, movement patterns, and methodological protocols. While inertial measurement units (IMUs) provide valid and reliable temporal data [28,29], differences in trial length and repetition number hinder comparability. To maximize reliability, our protocol averaged 15 repetitions across three trials, despite evidence that fewer repetitions could suffice for some exercises [29]. The participant sample was intentionally homogeneous (healthy young adults, balanced by sex and fitness level) to reduce the confounding effects of conditioning [15]. Despite methodological differences in the literature, our SMT values for EBM tasks—hopping, finger tapping, and walking—closely match previous reports of ~2 Hz [2,3,4,8,11,13,14,15,16,17,18,19,20,21]. For the PFE squat task, our observed SMT (0.49 Hz) is nearly identical to the 0.52 Hz reported by King and Hannan [32] for the same task. Hip and shoulder abduction SMTs partly align with those found by Peckel and colleagues [20], with slight differences most likely attributable to plane-of-motion effects. Specifically, Peckel and colleagues [20] reported a mean SMT of 0.75 Hz for hip oscillation in the sagittal plane (hip flexion-extension), compared to our observed 0.71 Hz for hip abduction. For shoulder movements, they found an SMT of 0.78 Hz for sagittal plane oscillation, while our shoulder abduction SMT was lower at 0.57 Hz. These differences likely reflect biomechanical variations related to movement planes. No directly comparable data were found for back extension, sit-up, push-up, or lunge. Overall, our task tempos (0.36–2.02 Hz) lie well within the 0.25–5 Hz range reported for human rhythmic movement [1,2,4,5,6,20,22,25,26].

Group-level variation in SMT was higher for PFE (avg CVgroup ~ 22.8%) than for EBM (~12.8%) tasks, reflecting more pronounced differences between individuals as task complexity and biomechanical demands increase. Sit-up and back extension approached CVgroup values of 25.3% and 33.8%, respectively—near the upper limit for group homogeneity [33]—suggesting stronger dependence on individual characteristics such as muscle strength and anthropometry. Within EBMs, walking exhibited very low group variation (CVgroup ~ 5.4%), suggesting a near-universal SMT, followed by hopping (CVgroup ~ 10.4%). Within EBM tasks, walking exhibited very low group variation (CVgroup ≈ 5.4%), indicating a near-universal SMT, followed by hopping (CVgroup ≈ 10.4%). Finger tapping showed higher group variation (CVgroup ≈ 22.6%), extending from exceptionally low SMT values around 0.3 Hz up to high values near 5 Hz. This finding aligns with previous literature reporting a broad SMT range [1,2,20] that challenges the notion of a single “universal” ≈ 2 Hz tempo for this task.

*Task Dependency of Temporal Structure.* The significant differences in rhythmic temporal structure among tasks closely mirror the observed variations in SMT. Specifically, the shorter tcycle and absolute phase durations (tphase1 and tphase2) found in EBM tasks align with their faster SMTs, highlighting rapid and efficient repetitions. Conversely, the longer durations observed in PFE tasks correspond to their slower SMTs, reflecting the greater muscular effort and control these exercises require, which demands more deliberate timing [1,20,27].

Yet, a different pattern emerges when considering relative phase durations (%tphase1 and %tphase2). Although these metrics vary significantly between tasks—indicating task dependency, they are not systematically associated with task type (EBM vs. PFE) but rather overlap across the EBM and PFE types. While EBM tasks differ from one another, some share similar relative timing with specific PFE tasks. For instance, walking and squat tasks exhibit nearly equal relative phase durations (~50% each). In contrast, hopping, hip abduction, back extension, sit-up, and shoulder abduction show slightly shorter %tphase1 and longer %tphase2 (≈48% and 52%, respectively), whereas finger tapping, push-up, and lunge display the opposite pattern (longer %tphase1 at ≈53% and shorter %tphase2 at ≈47%).

Overall, relative phase durations remain narrowly ranged (47–53%), indicating a remarkably consistent phase relationship close to 1:1, with each phase occupying roughly half the total cycle duration. This near-equal partitioning across tasks reflects a shared internal temporal framework across diverse motor tasks. For walking, the gait cycle—which includes two steps—is well documented to divide approximately 50–50%, with each step lasting about half the full gait cycle [34,35]. This fundamental rhythmic pattern serves as a canonical example of temporal organization in human movement. Our findings extend this principle: despite task-dependent differences in tempo and absolute durations, the internal temporal structure across all tasks remains close to this 1:1 ratio. This suggests that the central nervous system organizes rhythmically executed movements according to a common, robust timing mechanism—a shared internal clock—that transcends biomechanical complexity and tempo [17,36].

Supporting this view, group-level variation in relative phase durations was consistently low (≤7.2% across all tasks), averaging 4.8% for EBMs and 3.8% for PFEs. Such low group-level variation highlights a stable, shared coordination pattern across individuals. This suggests that, despite task-driven differences in absolute timing metrics, participants preserve a common temporal organization in executing rhythmic movements [37]. This group-level stability of relative timing likely reflects central neural control patterns that persist regardless of individual differences in muscular strength or range of motion. Thus, even though individuals may execute movements at different speeds, the fundamental internal temporal structure remains remarkably stable across the group.

The maintenance of a stable approximately 1:1 phase ratio reflects an inherent mechanism of motor control and performance. Kelso [38] demonstrated that during rhythmic movements, such as bimanual coordination, the in-phase pattern (1:1) represents the most stable form of coordination. When individuals attempt to maintain more complex or asymmetric rhythms at higher speeds, their movements spontaneously revert to this stable 1:1 ratio. A similar innate consistency of the 1:1 phase ratio has been reported across other motor tasks, where it emerges as a naturally spontaneous and robust neural state [39,40]. The observed tendency toward a 1:1 ratio indicates that this coordination pattern is not simply a matter of conscious choice but rather a natural and stable pattern of the human motor system—ideal for achieving smooth and efficient rhythmic movement [38,39,40,41].

*Task Dependency of Temporal Structure Stability.* Across all movement tasks, temporal structure stability (assessed through individual variability—both within- and between-trial—was consistently greater for relative compared to absolute durations. Specifically, relative durations showed an average within-trial variability of ≈3.1% and between-trial variability of ≈1.4%, whereas absolute durations exhibited higher variability (within-trial ≈ 4.4% and between-trial ≈ 4.7%). Functionally, this consistency may optimize movement efficiency, conserve energy, and maintain control during repetitive cyclical actions. As highlighted by these low variability values, and as noted in Section 4, the observed 1:1 pattern indicates that participants maintained a stable internal phase ratio—an inherently robust coordination state—even as absolute timing adapted to task-specific demands [38,39,40,41]. The lower variability in relative phases supports the view that central neural control prioritizes phase symmetry over absolute speed, even under varying biomechanical constraints. Instead, the slightly higher variability in absolute durations likely reflects necessary anticipatory adjustments and dynamic corrections to preserve the overall temporal organization [17].

Building on this stable temporal framework, temporal structure also revealed clear task dependency. Walking, a highly automatized EBM task, exhibited the lowest individual variability across all temporal metrics (≈1.9% within-trial; ≈0.9% between-trial), reflecting reliance on deeply embedded motor programs and spinal pattern generators [11,12]. Hopping also showed low individual variability, a finding consistent with well-practiced rhythmic patterns. In contrast, PFE tasks showed slightly higher individual variability, especially those involving substantial trunk motion (sit-up, back extension) or challenging postural stability (hip abduction, lunge). These demands introduce more degrees of freedom in motor control, necessitating the continuous fine-tuning of muscle activation and coordination. Even so, overall individual variability remained low: within-trial (avg CVind ≈ 3.9%) and between-trial (mean CVind ≈ 3.4%) values stayed consistently low, with no metric exceeding 7.8%. These results indicate that, regardless of slight CVind differences, the rhythmic temporal structure remained robust across the full range of movement tasks, allowing participants to execute movements precisely and repeatably even under more challenging biomechanical conditions [5,6,23].

Importantly, the control of movement tempo appears to be a highly accurate and prioritized aspect of rhythmic motor performance. This temporal structure stability does not stem solely from biomechanical properties such as motor tempo; rather, it reflects a robust neural control mechanism that sustains consistent timing even if other parameters fluctuate [10]. Every person seems to operate with a reliable internal timing mechanism—an “internal clock”—which fosters strong intra-individual consistency across repetitions and trials [1]. The observed low intra-individual variability, coupled with stable relative timing, may partly reflect history dependence on motor control: temporal characteristics from prior movement cycles influence the subsequent rhythmic output, enhancing temporal structure stability and enabling smooth transitions between cycles. This dynamic feedback mechanism allows the central nervous system to continuously recalibrate motor timing in response to internal and external perturbations.

Overall, the preservation of temporal structure stability—reflected in the low individual variability in both absolute (tcycle, tphase1, tphase2) and relative (%tphase1, %tphase2) durations—across both EBM and PFE tasks in the present sample indicates consistent rhythmic performance within individual trials and high repeatability between-trials. Thus, for the participants tested, movements were executed reliably, supporting the conclusion that the measured temporal metrics are robust and can be considered trustworthy indicators of spontaneous motor performance. Moreover, this stability reflects not only motor precision but also skill proficiency, as consistent temporal patterns are associated with reduced attentional demands and increased automaticity [23,42]. While task type influenced the magnitude of stability, the underlying control processes remained resilient, highlighting both a shared neural timing structure and individual adaptations to each movement’s mechanical demands.

*Practical Application.* The findings of this study offer valuable insights for practitioners in exercise science, rehabilitation, and sports training by emphasizing the importance of adapting rhythmic cues and movement tempos to the specific biomechanical demands of different activities [1,13,15,26]. For example, in clinical rehabilitation, the slower SMT values observed in PFE tasks may inform protocols for patients recovering from musculoskeletal injuries or postural control impairments, where deliberately slower tempos can enhance stability and reduce strain. Conversely, the faster and more automatic tempos observed in EBM tasks (e.g., walking or hopping) may be leveraged in gait rehabilitation, where rhythmic auditory stimulation or metronome cues have been shown to restore stable walking patterns in stroke and Parkinson’s disease patients [43,44]. In athletic training, tailoring training programs to an individual’s spontaneous motor tempo can enhance movement control and efficiency, potentially improving performance while reducing injury risk. Also, rhythmic cueing can be introduced to reinforce a consistent technique under fatigue, enhancing motor learning. Furthermore, inertial sensor systems provide a powerful tool for assessing temporal motor behavior in real-world settings, opening new opportunities for remote monitoring and personalized interventions [28,29].

*Limitations.* While this study offers novel insights into spontaneous motor performance, several limitations should be noted. First, the participant sample consisted exclusively of healthy young adults, which may limit the generalizability of the findings to populations with different fitness levels, ages, or clinical conditions. Although SMT tends to be a reliable individual measure over time, it does change across the lifespan [1], suggesting the need for broader demographic inclusion in future research. Second, although the chosen tasks represent a variety of physical fitness movements, they do not cover the full complexity of motor behaviors encountered in real-world or sport-specific environments. Future studies incorporating more dynamic, unpredictable, and ecologically valid tasks could provide a deeper understanding of how temporal structures adapt to diverse and complex conditions.

## 5. Conclusions

In conclusion, this study demonstrates that spontaneous motor performance is strongly task-dependent, with movement tempo (SMT) and absolute cycle and phase durations varying systematically across different rhythmic tasks. Established biomechanical model tasks (walking, hopping, finger tapping) elicited faster tempos and shorter absolute durations, while physical fitness-based exercises with higher biomechanical and postural demands showed slower tempos and longer durations. Despite these differences, relative phase durations remained remarkably stable across all tasks, consistently approximating a 1:1 ratio, highlighting a shared internal timing framework that organizes rhythmic movement. Temporal structure stability, assessed through within-trial and between-trial variability, was consistently low, indicating reliable and repeatable rhythmic performance across participants.

These findings advance our understanding of the central timing mechanisms that govern motor control, showing that internal temporal organization is robust across diverse movement contexts. While potential implications for training and rehabilitation are suggested, these applications remain speculative given this study’s healthy adult sample and laboratory-based setting. Future research should explore these patterns in broader populations and ecologically valid conditions to evaluate practical applications.

## Data Availability

The data are not publicly available due to ethical restrictions.

## Abbreviations

The following abbreviations are used in this manuscript:

SMT	Spontaneous motor tempo
EBM	Established biomechanical Model
PFEs	Physical fitness exercise-based tasks
WALK	Walking
HOP	Two-leg hop in place
FT	Finger tapping
SQR	Squat
LNG	Lunge
PU	Push-up
SU	Sit-up
BE	Back extension
SA	Shoulder abduction
HA	Hip abduction

## Appendix A. Mean ± SD and ANOVA Results

**Table A1:** **Temporal structure metrics.** Mean (SD) values of cycle duration (tcycle), absolute phase durations (tphase1, tphase2), and relative phase durations (%tphase1, %tphase2) for each movement task. ANOVA statistics (F, *p*, partial η^2^, and observed power) are reported for task effect.

	Temporal Structure Metrics				
	Mean (SD)				
	tcycle (s)	tphase1 (s)	tphase2 (s)	%tphase1 (%tcycle)	%tphase2 (%tcycle)
**EBM Tasks**					
Hopping	0.48 (0.05)	0.22 (0.02)	0.25 (0.04)	47.16 (3.4)	52.84 (3.4)
Finger Tapping	0.53 (0.12)	0.28 (0.07)	0.24 (0.06)	53.52 (3.16)	46.48 (3.16)
Walking (stride)	1.11 (0.06)	0.55 (0.03)	0.55 (0.03)	50.1 (0.57)	49.9 (0.57)
**PFE Tasks**					
Hip Abduction	1.47 (0.28)	0.7 (0.12)	0.77 (0.16)	47.64 (2.24)	52.36 (2.24)
Back Extension	1.51 (0.51)	0.73 (0.22)	0.78 (0.29)	48.59 (1.92)	51.41 (1.92)
Sit-Up	1.62 (0.41)	0.8 (0.2)	0.82 (0.22)	49.4 (1.98)	50.6 (1.98)
Push-Up	1.7 (0.34)	0.87 (0.19)	0.83 (0.16)	51.05 (1.12)	48.95 (1.12)
Shoulder Abduction	1.8 (0.33)	0.88 (0.15)	0.92 (0.19)	49.12 (1.56)	50.88 (1.56)
Squat	2.04 (0.37)	1.01 (0.19)	1.02 (0.19)	49.8 (2.23)	50.2 (2.23)
Lunge	2.74 (0.56)	1.46 (0.31)	1.28 (0.27)	53.33 (2.32)	46.67 (2.32)
**F-value**	158.94	177.50	126.11	28.04	28.04
***p*** **-value**	<0.001	<0.001	<0.001	<0.001	<0.001
**Partial η2**	0.85	0.86	0.82	0.50	0.50
**Observed Power**	1.00	1.00	1.00	1.00	1.00

EBM Tasks: established biomechanical models; PFE Tasks: physical fitness exercise-based tasks.

**Table A2:** **Within-trial individual variability (%CVind).** Mean (SD) values of %CVind, representing within-trial individual variability (fluctuations across repeated movement cycles), for cycle duration (tcycle), absolute phase durations (tphase1, tphase2), and relative phase durations (%tphase1, %tphase2) for each movement task. ANOVA statistics (F, *p*, partial η^2^, and observed power) are reported for task effect.

	Within-Trial Individual Variability (%CVind)				
	Mean (SD)				
	tcycle (%)	tphase1 (%)	tphase2 (%)	%tphase1 (%)	%tphase2 (%)
**EBM Tasks**					
Hopping	4.0 (1.3)	5.1 (2.7)	5.8 (2.1)	4.1 (1.9)	3.7 (2.2)
Finger Tapping	4.1 (1.3)	6.0 (1.9)	4.7 (1.7)	3.3 (1.1)	3.9 (1.5)
Walking (stride)	1.9 (0.6)	2.4 (0.6)	2.4 (0.6)	1.4 (0.3)	1.4 (0.3)
**PFE Tasks**					
Hip Abduction	4.3 (1.5)	5.1 (1.5)	6.3 (2.0)	3.9 (1.2)	3.5 (1.1)
Back Extension	4.6 (1.3)	7.5 (3.5)	6.6 (3.0)	5.5 (3.4)	5.2 (3.0)
Sit-Up	3.5 (1.1)	3.9 (1.5)	4.5 (1.5)	2.4 (1.2)	2.3 (1.1)
Push-Up	3.9 (1.7)	5.3 (3.5)	4.5 (2.6)	2.7 (1.9)	2.8 (1.9)
Shoulder Abduction	3.4 (1.0)	3.7 (1.2)	4.1 (1.2)	2.0 (0.9)	1.9 (0.7)
Squat	3.4 (1.1)	4.9 (1.5)	5.0 (1.6)	3.4 (1.5)	3.4 (1.5)
Lunge	3.1 (0.7)	4.0 (0.9)	3.9 (1.1)	2.4 (0.5)	2.7 (0.6)
**F-value**	14.47	15.05	14.68	17.44	15.67
***p*** **-value**	<0.001	<0.001	<0.001	<0.001	<0.001
**Partial η2**	0.34	0.35	0.34	0.38	0.36
**Observed Power**	1.00	1.00	1.00	1.00	1.00

EBM Tasks: established biomechanical models; PFE Tasks: physical fitness exercise-based tasks.

**Table A3:** **Between-trial individual variability (%CVind).** Mean (SD) values of %CVind, representing between-trial individual variability (fluctuations across repeated trials), for cycle duration (tcycle), absolute phase durations (tphase1, tphase2), and relative phase durations (%tphase1, %tphase2) for each movement task. ANOVA statistics (F, *p*, partial η^2^, and observed power) are reported for task effect.

	Between-Trial Individual Variability (%Cvind)				
	Mean (SD)				
	tcycle (%)	tphase1 (%)	tphase2 (%)	%tphase1 (%)	%tphase2 (%)
**EBM Tasks**					
Hopping	2.6 (1.5)	2.2 (1.9)	3.7 (2.5)	1.6 (2.1)	1.4 (1.8)
Finger Tapping	4.8 (3.1)	7.2 (5.3)	4.0 (3.4)	2.4 (2.3)	2.8 (2.5)
Walking (stride)	1.1 (0.9)	1.2 (0.9)	1.3 (0.9)	0.5 (0.3)	0.5 (0.3)
**PFE Tasks**					
Hip Abduction	4.6 (2.7)	4.2 (2.5)	5.3 (3.2)	1.5 (0.9)	1.4 (0.8)
Back Extension	5.5 (3.4)	5.0 (3.4)	6.3 (4.2)	1.8 (2.0)	1.7 (1.8)
Sit-Up	6.0 (6.1)	6.5 (7.2)	7.8 (8.8)	1.6 (2.6)	1.5 (2.4)
Push-Up	4.8 (3.3)	4.9 (2.6)	5.1 (4.3)	1.0 (1.5)	1.1 (1.5)
Shoulder Abduction	6.5 (5.8)	6.1 (5.4)	7.1 (6.2)	1.0 (1.0)	0.9 (1.0)
Squat	3.7 (2.9)	4.1 (2.9)	4.4 (3.4)	1.7 (1.9)	1.6 (1.7)
Lunge	4.8 (5.1)	5.4 (5.6)	4.5 (4.7)	1.3 (1.0)	1.4 (1.2)
**F-value**	5.73	6.21	5.06	2.84	4.04
***p*** **-value**	<0.001	<0.001	0.00	0.02	0.00
**Partial η2**	0.17	0.18	0.15	0.09	0.13
**Observed Power**	0.99	0.99	0.97	0.79	0.92

EBM Tasks: established biomechanical models; PFE Tasks: physical fitness exercise-based tasks.

## Appendix B. Pairwise Comparisons

**Table A4:** **Temporal structure metrics—pairwise comparisons.** *p*-values from pairwise comparisons for cycle duration (tcycle), absolute phase durations (tphase1, tphase2), and relative phase durations (%tphase1, %tphase2) between all movement tasks. Statistically significant differences are indicated (*p* < 0.05, Bonferroni-corrected). HOP: hopping; FT: finger tapping; WALK: walking; HA: hip abduction; BE: back extension; SU: sit-up; PU: push-up; SA: shoulder abduction; SQR: squat; LGN: lunge.

	*p*-Values of Temporal Structure Metrics									
	Task	HOP	FT	WALK	HA	BE	SU	PU	SA	SQR
**tcycle**	**FT**	1.000	—							
	**WALK**	0.000	0.000	—						
	**HA**	0.000	0.000	0.000	—					
	**BE**	0.000	0.000	0.010	0.057	—				
	**SU**	0.000	0.000	0.000	1.000	0.360	—			
	**PU**	0.000	0.000	0.000	0.073	0.471	1.000	—		
	**SA**	0.000	0.000	0.000	0.004	0.360	0.602	1.000	—	
	**SQR**	0.000	0.000	0.000	0.000	0.000	0.000	0.000	0.238	—
	**LNG**	0.000	0.000	0.000	0.000	0.000	0.000	0.000	0.000	0.000
**tphase1**	**FT**	0.001	—							
	**WALK**	0.000	0.000	—						
	**HA**	0.000	0.000	0.000	—					
	**BE**	0.000	0.000	0.012	0.010	—				
	**SU**	0.000	0.000	0.000	0.575	0.002	—			
	**PU**	0.000	0.000	0.000	0.001	0.002	0.522	—		
	**SA**	0.000	0.000	0.000	0.000	0.002	0.697	1.000	—	
	**SQR**	0.000	0.000	0.000	0.000	0.000	0.000	0.002	0.053	—
	**LNG**	0.000	0.000	0.000	0.000	0.000	0.000	0.000	0.000	0.000
**tphase2**	**FT**	1.000	—							
	**WALK**	0.000	0.000	—						
	**HA**	0.000	0.000	0.000	—					
	**BE**	0.000	0.000	0.010	0.284	—				
	**SU**	0.000	0.000	0.000	1.000	1.000	—			
	**PU**	0.000	0.000	0.000	1.000	1.000	1.000	—		
	**SA**	0.000	0.000	0.000	0.079	1.000	0.771	0.630	—	
	**SQR**	0.000	0.000	0.000	0.000	0.000	0.001	0.000	1.000	—
	**LNG**	0.000	0.000	0.000	0.000	0.000	0.000	0.000	0.000	0.000
**%tphase1 and** **%tphase2**	**FT**	0.000	—							
	**WALK**	0.003	0.000	—						
	**HA**	1.000	0.000	0.000	—					
	**BE**	1.000	0.000	0.002	1.000	—				
	**SU**	0.134	0.000	1.000	0.131	1.000	—			
	**PU**	0.000	0.043	0.035	0.000	0.001	0.022	—		
	**SA**	0.407	0.000	0.243	0.284	1.000	1.000	0.001	—	
	**SQR**	0.078	0.001	1.000	0.069	0.725	1.000	0.435	1.000	—
	**LNG**	0.000	1.000	0.000	0.000	0.000	0.000	0.002	0.000	0.000

**Table A5:** **Within-trial individual variability (%CVind)—pairwise comparisons.** *p*-values from pairwise comparisons of within-trial individual variability (%CVind) for cycle duration (tcycle), absolute phase durations (tphase1, tphase2), and relative phase durations (%tphase1, %tphase2) between all movement tasks. Statistically significant differences are indicated (*p* < 0.05, Bonferroni-corrected). HOP: hopping; FT: finger tapping; WALK: walking; HA: hip abduction; BE: back extension; SU: sit-up; PU: push-up; SA: shoulder abduction; SQR: squat; LGN: lunge.

	*p*-Values of Within-Trial Individual Variability									
	Task	HOP	FT	WALK	HA	BE	SU	PU	SA	SQR
**tcycle**	**FT**	1.000	—							
	**WALK**	0.000	0.000	—						
	**HA**	1.000	1.000	0.000	—					
	**BE**	1.000	1.000	0.000	0.010	—				
	**SU**	1.000	1.000	0.000	0.450	0.020	—			
	**PU**	1.000	1.000	0.000	1.000	1.000	1.000	—		
	**SA**	0.530	0.090	0.000	0.030	0.020	1.000	1.000	—	
	**SQR**	1.000	0.980	0.000	0.070	0.010	1.000	1.000	1.000	—
	**LNG**	0.040	0.030	0.000	0.000	0.000	1.000	0.370	1.000	1.000
**tphase1**	**FT**	1.000	—							
	**WALK**	0.000	0.000	—						
	**HA**	1.000	1.000	0.000	—					
	**BE**	0.190	1.000	0.000	0.000	—				
	**SU**	1.000	0.000	0.000	0.320	0.000	—			
	**PU**	1.000	1.000	0.000	1.000	0.390	1.000	—		
	**SA**	0.270	0.000	0.000	0.010	0.000	1.000	0.960	—	
	**SQR**	1.000	0.530	0.000	1.000	0.020	0.580	1.000	0.130	—
	**LNG**	1.000	0.000	0.000	0.040	0.000	1.000	1.000	1.000	0.310
**tphase2**	**FT**	0.370	—							
	**WALK**	0.000	0.000	—						
	**HA**	1.000	0.110	0.000	—					
	**BE**	1.000	0.250	0.000	0.010	—				
	**SU**	0.530	1.000	0.000	0.010	0.070	—			
	**PU**	1.000	1.000	0.000	0.020	0.090	1.000	—		
	**SA**	0.000	1.000	0.000	0.000	0.070	1.000	1.000	—	
	**SQR**	1.000	1.000	0.000	0.240	0.280	1.000	1.000	1.000	—
	**LNG**	0.000	1.000	0.000	0.000	0.000	1.000	1.000	1.000	0.170
**%tphase1** **and** **%tphase2**	**FT**	1.000	—							
	**WALK**	0.000	0.000	—						
	**HA**	1.000	1.000	0.000	—					
	**BE**	1.000	0.140	0.000	0.000	—				
	**SU**	0.010	0.130	0.000	0.000	0.000	—			
	**PU**	0.450	1.000	0.020	0.200	0.010	1.000	—		
	**SA**	0.000	0.000	0.030	0.000	0.000	1.000	1.000	—	
	**SQR**	1.000	1.000	0.000	1.000	0.090	0.080	1.000	0.000	—
	**LNG**	0.000	0.020	0.000	0.000	0.000	1.000	1.000	1.000	0.060

**Table A6:** **Between-trial individual variability (%CVind)—pairwise comparisons.** *p*-values from pairwise comparisons of between-trial individual variability (%CVind) for cycle duration (tcycle), absolute phase durations (tphase1, tphase2), and relative phase durations (%tphase1, %tphase2) between all movement tasks. Statistically significant differences are indicated (*p* < 0.05, Bonferroni-corrected). HOP: hopping; FT: finger tapping; WALK: walking; HA: hip abduction; BE: back extension; SU: sit-up; PU: push-up; SA: shoulder abduction; SQR: squat; LGN: lunge.

	*p*-Values of Between-Trial Individual Variability									
	Task	HOP	FT	WALK	HA	BE	SU	PU	SA	SQR
**tcycle**	**FT**	0.001	—							
	**WALK**	0.000	0.000	—						
	**HA**	0.000	0.755	0.000	—					
	**BE**	0.000	0.431	0.000	0.317	—				
	**SU**	0.007	0.322	0.000	0.256	0.625	—			
	**PU**	0.004	0.948	0.000	0.818	0.453	0.289	—		
	**SA**	0.002	0.172	0.000	0.143	0.625	0.746	0.230	—	
	**SQR**	0.099	0.181	0.000	0.163	0.036	0.046	0.182	0.030	—
	**LNG**	0.029	0.977	0.001	0.862	0.458	0.304	0.990	0.090	0.240
**tphase1**	**FT**	0.000	—							
	**WALK**	0.017	0.000	—						
	**HA**	0.000	0.005	0.000	—					
	**BE**	0.000	0.078	0.000	0.240	—				
	**SU**	0.005	0.721	0.000	0.113	0.233	—			
	**PU**	0.000	0.025	0.000	0.304	0.864	0.231	—		
	**SA**	0.001	0.474	0.000	0.099	0.233	0.804	0.326	—	
	**SQR**	0.009	0.017	0.000	0.904	0.170	0.072	0.272	0.109	—
	**LNG**	0.005	0.242	0.001	0.286	0.674	0.406	0.685	0.456	0.270
**tphase2**	**FT**	0.565	—							
	**WALK**	0.000	0.000	—						
	**HA**	0.036	0.155	0.000	—					
	**BE**	0.010	0.035	0.000	0.531	—				
	**SU**	0.027	0.045	0.000	0.163	0.385	—			
	**PU**	0.148	0.292	0.000	0.859	0.297	0.123	—		
	**SA**	0.015	0.027	0.000	0.199	0.385	0.688	0.206	—	
	**SQR**	0.418	0.758	0.000	0.303	0.040	0.050	0.486	0.049	—
	**LNG**	0.438	0.672	0.001	0.499	0.079	0.033	0.637	0.014	0.883
**%tphase1** **and** **%tphase2**	**FT**	0.141	—							
	**WALK**	0.004	0.000	—						
	**HA**	0.715	0.066	0.000	—					
	**BE**	0.758	0.383	0.001	0.052	—				
	**SU**	0.999	0.322	0.023	0.796	0.678	—			
	**PU**	0.229	0.015	0.044	0.139	0.111	0.284	—		
	**SA**	0.136	0.008	0.024	0.074	0.678	0.121	0.839	—	
	**SQR**	0.967	0.246	0.004	0.698	0.680	0.957	0.207	0.112	—
	**LNG**	0.402	0.022	0.001	0.354	0.187	0.440	0.460	0.311	0.329

## Appendix D

**Table A7:** Intraclass Correlation Coefficients (ICCs) with 95% Confidence Intervals. This table presents the test–retest reliability of cycle duration (tcycle), absolute phase durations (tphase1, tphase2), and relative phase durations (%tphase1, %tphase2) for each movement task. Values are reported as Intraclass Correlation Coefficients (ICCs) with corresponding 95% Confidence Intervals (CIs). ICC values closer to 1.0 indicate higher reliability.

	Temporal Structure Metrics				
	tcycle	tphase1	tphase2	%tphase1	%tphase2
	ICC (95% CI)	ICC (95% CI)	ICC (95% CI)	ICC (95% CI)	ICC (95% CI)
HOP	0.997 (0.995–0.998)	0.985 (0.976–0.992)	0.997 (0.995–0.998)	0.990 (0.984–0.994)	0.990 (0.984–0.994)
FT	0.999 (0.998–0.999)	0.998 (0.997–0.999)	0.999 (0.998–0.999)	0.992 (0.987–0.996)	0.992 (0.987–0.996)
WALK	0.994 (0.990–0.997)	0.992 (0.986–0.995)	0.993 (0.989–0.996)	0.955 (0.928–0.975)	0.955 (0.928–0.975)
BE	0.999 (0.999–1.000)	0.998 (0.997–0.999)	0.999 (0.999–1.000)	0.947 (0.915–0.971)	0.947 (0.915–0.971)
HA	0.998 (0.997–0.999)	0.997 (0.996–0.999)	0.997 (0.995–0.998)	0.980 (0.968–0.989)	0.980 (0.968–0.989)
SU	0.999 (0.999–1.000)	0.999 (0.998–0.999)	0.999 (0.998–0.999)	0.988 (0.981–0.994)	0.988 (0.981–0.994)
PU	0.997 (0.995–0.998)	0.996 (0.993–0.998)	0.994 (0.991–0.997)	0.903 (0.841–0.948)	0.903 (0.841–0.948)
SA	0.999 (0.998–0.999)	0.998 (0.997–0.999)	0.999 (0.998–0.999)	0.989 (0.982–0.994)	0.989 (0.982–0.994)
SQR	0.999 (0.998–0.999)	0.998 (0.996–0.999)	0.997 (0.996–0.999)	0.981 (0.970–0.990)	0.981 (0.970–0.990)
LNG	0.999 (0.999–1.000)	0.999 (0.998–0.999)	0.999 (0.998–0.999)	0.991 (0.986–0.995)	0.991 (0.986–0.995)

ICC: Excellent (≥0.75), Moderate to Good (0.40–0.75), Poor (<0.40) [45]).

**Table A8:** **Standard Error of Measurement (SEM and SEM%).** This table presents the Standard Error of Measurement (SEM) and SEM expressed as a percentage of the mean (SEM%) of cycle duration (tcycle), absolute phase durations (tphase1, tphase2), and relative phase durations (%tphase1, %tphase2) for each movement task. Lower SEM and SEM% values reflect less measurement error and greater consistency in repeated assessments.

	Temporal Structure Metrics									
	tcycle		tphase1		tphase2		%tphase1		%tphase2	
	SEM	SEM%	SEM	SEM%	SEM	SEM%	SEM	SEM%	SEM	SEM%
HOP	0.01	2.41	0.01	3.47	0.01	3.63	1.32	2.81	1.32	2.51
FT	0.01	2.83	0.01	3.88	0.01	3.40	1.10	2.05	1.10	2.36
WALK	0.02	1.43	0.01	1.77	0.01	1.65	0.47	0.93	0.47	0.93
SQR	0.05	3.13	0.03	4.63	0.03	4.02	1.70	3.49	1.70	3.30
LNG	0.04	2.88	0.02	3.40	0.03	4.26	1.21	2.55	1.21	2.32
PU	0.05	2.78	0.03	3.45	0.03	3.58	0.83	1.68	0.83	1.64
SU	0.06	3.39	0.04	4.26	0.03	4.12	1.05	2.06	1.05	2.15
BE	0.04	2.41	0.02	2.77	0.03	2.93	0.64	1.30	0.64	1.26
SA	0.05	2.47	0.03	3.40	0.04	3.66	1.17	2.36	1.17	2.34
HA	0.06	2.27	0.04	2.82	0.04	2.97	0.82	1.54	0.82	1.75

SEM%: Low (≤10%), Acceptable (10–15%), High (>15%) [29].

**Table A9:** **Minimal Detectable Change at 95% Confidence (MDC95 and MDC95%).** This table presents the Minimal Detectable Change (MDC95) and MDC95 expressed as a percentage of the mean (MDC95%) of cycle duration (tcycle), absolute phase durations (tphase1, tphase2), and relative phase durations (%tphase1, %tphase2) for each movement task. MDC95 represents the smallest change that can be interpreted as a real difference beyond measurement error, with 95% confidence. Lower MDC95 and MDC95% values indicate higher practical reliability and greater sensitivity to detect real changes over time.

	Temporal Structure Metrics									
	tcycle		tphase1		tphase2		%tphase1		%tphase2	
	MDC	MDC%	MDC	MDC%	MDC	MDC%	MDC	MDC%	MDC	MDC%
HOP	0.03	5.62	0.02	8.10	0.02	8.47	3.09	6.55	3.09	5.85
FT	0.03	6.59	0.03	9.05	0.02	7.94	2.56	4.78	2.56	5.50
WALK	0.04	3.34	0.02	4.12	0.02	3.84	1.09	2.17	1.09	2.18
SQR	0.11	7.31	0.08	10.81	0.07	9.39	3.96	8.14	3.96	7.70
LNG	0.10	6.72	0.06	7.94	0.08	9.94	2.83	5.94	2.83	5.41
PU	0.11	6.48	0.06	8.05	0.07	8.34	1.93	3.91	1.93	3.82
SU	0.13	7.90	0.09	9.94	0.08	9.61	2.45	4.81	2.45	5.01
BE	0.10	5.62	0.06	6.46	0.06	6.83	1.49	3.04	1.49	2.93
SA	0.12	5.76	0.08	7.93	0.09	8.54	2.74	5.50	2.74	5.46
HA	0.15	5.29	0.10	6.59	0.09	6.92	1.91	3.58	1.91	4.09

## References

[1] Desbernats A., Martín E., Tallet J. (2023). Which Factors Modulate Spontaneous Motor Tempo? A Systematic Review of Literature. Front. Psychol..

[2] Hammerschmidt D., Frieler K., Wöllner C. (2021). Spontaneous Motor Tempo: Investigating Psychological, Chronobiological, and Demographic Factors in a Large-Scale Online Tapping Experiment. Front. Psychol..

[3] Rose D., Cameron D.J., Lovatt P., Grahn J.A., Annett L.E. (2020). Comparison of Spontaneous Motor Tempo during Finger Tapping, Toe Tapping and Stepping on the Spot in People with and without Parkinson’s Disease. J. Mov. Disord..

[4] Rose D., Ott L., Guérin S.M., Annett L.E., Lovatt P., Delevoye-Turrell Y.N. (2021). A General Procedure to Measure the Pacing of Body Movements Timed to Music and Metronome in Younger and Older Adults. Sci. Rep..

[5] Smoll F.L. (1975). Preferred Tempo in Performance of Repetitive Movements. Percept. Mot. Ski..

[6] Smoll F.L. (1975). Between-Days Consistency in Personal Tempo. Percept. Mot. Ski..

[7] Engler B.H., Zamm A., Møller C. (2024). Spontaneous Rates Exhibit High Intra-Individual Stability across Movements Involving Different Biomechanical Systems and Cognitive Demands. Sci. Rep..

[8] Fraisse P., Deutsch D. (1982). Rhythm and Tempo. The Psychology of Music.

[9] Repp B.H., Su Y.H. (2013). Sensorimotor Synchronization: A Review of Recent Research (2006–2012). Psychon. Bull. Rev..

[10] Hansen E.A. (2015). On Voluntary Rhythmic Leg Movement Behaviour and Control during Pedalling. Acta Physiol..

[11] MacDougall H.G., Moore S.T. (2005). Marching to the Beat of the Same Drummer: The Spontaneous Tempo of Human Locomotion. J. Appl. Physiol..

[12] Rose D., Delevoye-Turrell Y., Ott L., Annett L.E., Lovatt P.J. (2019). Music and Metronomes Differentially Impact Motor Timing in People with and without Parkinson’s Disease: Effects of Slow, Medium, and Fast Tempi on Entrainment and Synchronization Performances in Finger Tapping, Toe Tapping, and Stepping on the Spot Tasks. Park. Dis..

[13] Repp B.H. (2005). Sensorimotor Synchronization: A Review of the Tapping Literature. Psychon. Bull. Rev..

[14] Moelants D. Preferred Tempo Reconsidered. Proceedings of the 7th International Conference on Music Perception and Cognition.

[15] Delevoye-Turrell Y., Dione M., Agneray G. (2014). Spontaneous Motor Tempo Is the Easiest Pace to Act Upon for Both the Emergent and the Predictive Timing Modes. Procedia Soc. Behav. Sci..

[16] Collyer C.E., Broadbent H.A., Church R.M. (1994). Preferred Rates of Repetitive Tapping and Categorical Time Production. Percept. Psychophys..

[17] Rousanoglou E., Boudolos K. (2006). Rhythmic Performance during a Whole Body Movement: Dynamic Analysis of Force–Time Curves. Hum. Mov. Sci..

[18] Van Noorden L., Moelants D. (1999). Resonance in the Perception of Musical Pulse. J. New Music Res..

[19] Vanneste V., Pouthas J.H., Wearden J. (2001). Temporal Control of Rhythmic Performance: A Comparison between Young and Old Adults. Exp. Aging Res..

[20] Peckel M., Pozzo T., Bigand E. (2014). The Impact of the Perception of Rhythmic Music on Self-Paced Oscillatory Movements. Front. Psychol..

[21] Styns F., Van Noorden L., Moelants D., Leman M. (2007). Walking on Music. Hum. Mov. Sci..

[22] Hammerschmidt D., Wöllner C. (2023). Spontaneous Motor Tempo over the Course of a Week: The Role of the Time of the Day, Chronotype, and Arousal. Psychol. Res..

[23] Stergiou N., Decker L.M. (2011). Human Movement Variability, Nonlinear Dynamics, and Pathology: Is There a Connection?. Hum. Mov. Sci..

[24] Varlet M., Williams R.A., Keller P.E. (2018). Effects of Pitch and Tempo of Auditory Rhythms on Spontaneous Movement Entrainment and Stabilisation. Psychol. Res..

[25] Emmanouil A., Rousanoglou E., Georgaki A., Boudolos K. (2021). When Musical Accompaniment Allows the Preferred Spatio-Temporal Pattern of Movement. Sports Med. Int. Open.

[26] Coste A., Salesse R.N., Gueugnon M., Marín L., Bardy B.G. (2018). Standing or Swaying to the Beat: Discrete Auditory Rhythms Entrain Stance and Promote Postural Coordination Stability. Gait Posture.

[27] Burger B., Thompson M., Luck G., Saarikallio S., Toiviainen P. (2013). Influences of Rhythm- and Timbre-Related Musical Features on Characteristics of Music-Induced Movement. Front. Psychol..

[28] Cudejko T., Button K., Al-Amri M. (2022). Validity and Reliability of Accelerations and Orientations Measured Using Wearable Sensors during Functional Activities. Sci. Rep..

[29] Emmanouil A., Rousanoglou E., Boudolos K. (2024). Two Repetitions May Be Enough! Reliability of Movement Timing in Physical Fitness Exercises Performed by Young, Trained Adults Using Inertial Sensors. Biomechanics.

[30] Field A. (2018). Discovering Statistics Using IBM SPSS Statistics.

[31] Cohen J. (1988). Statistical Power Analysis for the Behavioral Sciences.

[32] King A.C., Hannan K.B. (2019). Segment Coordination Variability during Double Leg Bodyweight Squats at Different Tempos. Int. J. Sports Med..

[33] Hopkins W.G. (2000). Measures of Reliability in Sports Medicine and Science. Sports Med..

[34] Iosa M., Fusco A., Marchetti F., Morone G., Caltagirone C., Paolucci S., Peppe A. (2013). The Golden Ratio of Gait Harmony: Repetitive Proportions of Repetitive Gait Phases. BioMed Res. Int..

[35] Winter D.A., Sienko S.E. (1988). Biomechanics of Below-Knee Amputee Gait. J. Biomech..

[36] Ivry R.B., Schlerf J.E. (2008). Dedicated and Intrinsic Models of Time Perception. Trends Cogn. Sci..

[37] Raffalt P.C., Alkjær T., Simonsen E.B. (2016). Intra- and Inter-Subject Variation in Lower Limb Coordination during Countermovement Jumps in Children and Adults. Hum. Mov. Sci..

[38] Kelso J.A.S. (1984). Phase Transitions and Critical Behavior in Human Bimanual Coordination. Am. J. Physiol.-Regul. Integr. Comp. Physiol..

[39] Kelso J.A.S., Zanone P.G. (2002). Coordination Dynamics of Learning and Transfer across Different Effector Systems. J. Exp. Psychol. Hum. Percept. Perform..

[40] Schmidt R.C., Turvey M.T. (1994). Phase-Entrainment Dynamics of Visually Coupled Rhythmic Movements. Biol. Cybern..

[41] Wilson A.D., Collins D.R., Bingham G.P. (2005). Perceptual Coupling in Rhythmic Movement Coordination: Stable Perception Leads to Stable Action. Exp. Brain Res..

[42] Levy-Tzedek S., Ben Tov M., Karniel A. (2011). Rhythmic Movements are Larger and Faster but with the Same Frequency on Removal of Visual Feedback. J. Neurophysiol..

[43] Thaut M.H., McIntosh G.C., Rice R.R. (1996). Rhythmic facilitation of gait training in hemiparetic stroke rehabilitation. J. Neurol. Sci..

[44] Nombela C., Hughes L.E., Owen A.M., Grahn J.A. (2013). Into the groove: Can rhythm influence Parkinson’s disease?. Neurosci. Biobehav. Rev..

[45] Fleiss J.L. (1986). The Design and Analysis of Clinical Experiments.

